# Learning from patient involvement in a clinical study analyzing PET/CT in women with advanced breast cancer

**DOI:** 10.1186/s40900-019-0174-y

**Published:** 2020-01-06

**Authors:** Marianne Vogsen, Susanne Geneser, Marie Lykke Rasmussen, Mogens Hørder, Malene Grubbe Hildebrandt

**Affiliations:** 1 Department of Nuclear Medicine, Odense University Hospital, Kloevervaenget 47, DK-5000 Odense, Denmark; 2 Department of Oncology, Odense University Hospital, Odense, Denmark; 3 Department of Clinical Research, University of Southern Denmark, Odense, Denmark; 4 Centre for Personalized Response Monitoring in Oncology (PREMIO), Odense University Hospital, Odense, Denmark; 5 Patient and public representative, Danish Breast Cancer Patient Organization (DBO), Odense, Denmark; 6 Department of Public Health, University of Southern Denmark, Odense, Denmark; 7 Centre for Innovative Medical Technology, CIMT, Odense University Hospital, Odense, Denmark

**Keywords:** PPI, Patient and public involvement in research, Lived experience, Advanced breast cancer, PET/CT

## Abstract

**Background:**

Despite increasing interest in patient involvement in health care research, researchers may be uncertain about the benefits of involving patients in the design and conduction of clinical studies. We aimed to evaluate the impact of patient involvement on patient recruitment and retention in a clinical study of PET/CT in women with advanced breast cancer. Further, we report our experience regarding the researchers’ attitudes towards involving patients as partners in the research process.

**Methods:**

Two patient representatives from the Danish Breast Cancer Organization were invited as partners in the research team. These patient partners were asked to contribute in particular to participator information material and evaluation of ethical aspects of the study. The impact of patient involvement on patient recruitment was evaluated by comparing expected versus actual number of patients recruited, and then relating it to patient recruitment in a similar study at the same institution that did not involve patients as research partners.

**Results:**

Having patients as partners in the research team led to a major revision of the participator information material and improved patient recruitment. The expected number of patients was 260, but 380 were actually enrolled within the planned study period, thus 146% of the expected patient recruitment. In the previous study, only 100 of the expected 150 patients were enrolled during a 10-month extended study period, i.e. 67% of the expected number. Patient retention in the current study was high, with 86% of eligible patients attending follow-up scans. We observed initial resistance amongst researchers against inviting patients as team partners. This resistance gradually lessened during the study, and the most reluctant researchers at the beginning of the study later applauded the collaboration and the ideas generated by the patient representatives.

**Conclusion:**

Involving patients as partners in the research team resulted in major changes to the participator information material and contributed to higher than expected patient recruitment and retention. Furthermore, we observed a positive change of attitude amongst the researchers towards patient involvement in the research process.

**Trial registration:**

Ongoing study: ClinicalTrials.gov (NCT03358589).

Previous study: ClinicalTrials.gov (NCT01552655).

## Plain English summary

In this article, we share our experiences of involving patients as research partners in a clinical study of women with incurable breast cancer. Patient recruitment and retention were higher than expected in the study, and we observed a positive change of attitude in our research team towards patient involvement in the research process.

Two patients with previous experience of breast cancer were invited to be partners in the research team. They were asked to contribute in particular to participator information material and evaluation of ethical aspects of the study.

The patient partners suggested major revisions to the participator information material and contributed to a more patient-friendly enrollment process. This led to a higher number of patients being recruited than we expected. In contrast, a previous study that did not have patients in the research team had enrolled fewer patients than expected. The current study also had a high number of patients (86%) who continued to attend for regular follow-up scans.

We noted an initial resistance among researchers of the research team against inviting patients as partners, but we found that this gradually resolved over the course of the study.

Based on the results of this study, we will be inviting patients to be our research partners in more of our future clinical studies. We recommend that other researchers also consider doing this to ensure consideration of the patient perspective in study design and implementation.

## Background

Patients and members of the public have an important role in health care research due to their lived experience of the condition under study [1]. Involving patients as partners in a research team (PPI) aims at improving the quality of research by ensuring that more patient-relevant issues and endpoints are addressed [2]. The best effect of PPI is assumed to be achieved when patient representatives are included at the outset of the research process and throughout the whole research project [3]. The impact of PPI on patient recruitment seems greatest if the patient representatives have lived experience of the health condition under study [4].

There is an increasing interest in the impact of involving patients as partners in clinical studies, although PPI appears to be more common in qualitative than quantitative research [4, 5]. A recent systematic review and meta-analysis [4] and two previous observational studies [6, 7] showed a positive association between PPI and patient recruitment in clinical studies. The positive impact is suggested to be related to improved wording of written information, more effective ways of identifying study participants, and more patient-centered outcomes. Furthermore, PPI can lead to greater likelihood of study implementation and dissemination of research results [5, 8]. PPI is also suggested to have an impact on participant retention although only few studies have reported on this, and no significant effect has yet been shown [4].

Implementation of PPI in health care research may over time have an impact on the researchers, resulting in more positive attitudes towards patients as research partners. For the researcher, however, the impact of PPI in a study is difficult to estimate beforehand. As stated by Kristina Staley, “at the beginning of any research project, the researchers don’t know what they don’t know until they have involved the patients” [9–11].

Our aim was to evaluate the impact on patient recruitment and retention of having patients as research partners in a clinical study of breast cancer. The clinical study is a large, ongoing study investigating response monitoring by positron emission tomography/computed tomography (PET/CT) in women with advanced breast cancer. Our main outcome was the success rate for patient recruitment in the current study compared to that in a previous similar study that did not include patients in the research team. We also report our experience regarding researchers’ attitudes towards involving patients as partners in the research process.

## Methods

This is a descriptive report that is based on health care professionals’ experiences of involving patients as research partners in an ongoing clinical study. We compare our results to those of a previous clinical study that did not involve patients as research partners. The GRIPP-2 reporting guideline [12] was used to report these experiences.

### Previous clinical study without PPI

In a previous study from our department at Odense University Hospital, the use of PET/CT was compared with the standard breast cancer recurrence examination program of computed tomography (CT) scan and bone scintigraphy (NCT01552655) [13]. In this study, 100 women with previous breast cancer underwent CT, bone scintigraphy, and PET/CT, where the PET/CT set-up required two scans per day for the enrolled patients. Patients underwent a biopsy if advanced breast cancer was suspected. We found that PET/CT had higher accuracy than CT and bone scintigraphy, and it was thus implemented as the standard examination for breast cancer recurrence at our institution. No patient representatives were involved in the research team of this study.

### Current clinical study with PPI

The current study is a large, ongoing collaborative study of breast cancer patients at Odense University Hospital that was initiated in September 2017 (NCT03358589). The data on patient recruitment and retention that are reported here were from November 2019, i.e. 26 months after study initiation. At that time, the study was expected to comprise 260 women with suspected advanced breast cancer.

The study population is similar to that of the previous study (i.e. women with suspected metastatic spread from breast cancer), the only difference being that women with primary breast cancer with high risk of metastatic spread at the time of diagnosis were excluded from the previous study.

In the current study, women had no further scan if no metastases were detected on PET/CT, but all patients were asked to have a blood test for genomic mutations. This meant that the participator information material was quite complex due to the risk of incidental findings in the genome. If bone metastases were detected on PET/CT, the patient proceeded to whole-body MRI scan. If advanced breast cancer was suspected at PET/CT, the patient underwent a biopsy from a metastatic lesion to enable exact diagnosis and appropriate choice of treatment.

Advanced breast cancer is an incurable disease with a need for life-long medical treatment, e.g. chemotherapy. Women with biopsy-verified advanced breast cancer proceeded to the response monitoring stage of the study, where the treatment effect was evaluated every 3 months using standard CT and blinded PET/CT for research purposes.

### Patients as research partners

We invited previous breast cancer patients who were members of the Danish Breast Cancer Patient Organization to be partners in our research team. The Danish Breast Cancer Patient Organization is a large and established group of volunteers who have all previously had breast cancer. Two women responded within one week; their role in the organization was to arrange patient information meetings on patient-relevant subjects and to serve as patient consultants. Both women were involved in research councils at Odense University Hospital and in other breast cancer committees in Denmark. We considered these patients to have the physical and psychological resources to participate in our research group, and both women were invited as partners.

**Fig. 1:**
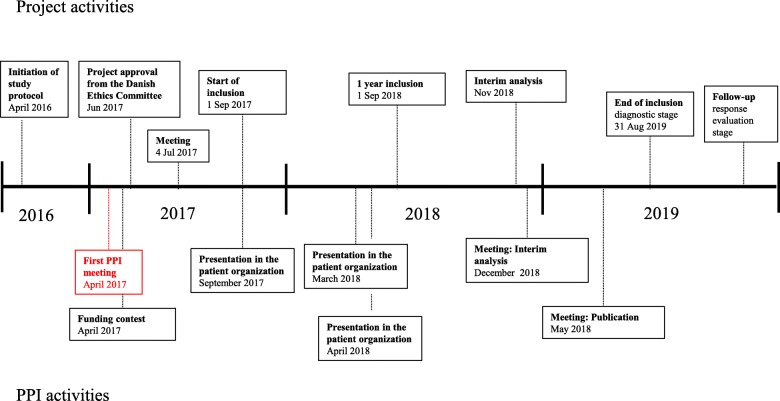
Diagram showing the timing of project activities and patient and public involvement (PPI) activities in an ongoing study of PET/CT in advanced breast cancer. Odense University Hospital, 2017–2019

These patient representatives were not provided with any training but were paid an acknowledgment fee that covered their transport expenses according to Danish standards. Both patients have contributed to the present manuscript. Throughout the study, ad hoc meetings were arranged in a smaller group consisting of two researchers (MV and MGH) and the two patient representatives (MLR and SG). These meetings were held at the Department of Nuclear Medicine at Odense University Hospital, and the timing and agendas are shown in Fig. 1.

Specific tasks for the patients in the PPI process were established before meeting the patient representatives. These tasks included: development of participator information material and written consent forms, active involvement in funding activities, interpretation of interim analysis, and dissemination of research results.

#### Development of written participator information material

Before the first meeting in April 2017, the study protocol and written participator information material were approved by the Danish Ethics Committee after small changes had been made (e.g. a study flowchart was requested). This written material was sent to the patient representatives for oral feedback; hence, no written material was expected from the patients. Further revision of the written material was arranged by e-mail, and it received final approval from the Danish Ethics Committee in June 2017.

#### Involvement in funding activities

In 2017, the University of Southern Denmark, Odense University Hospital, and the local television arranged a funding contest to improve the public’s knowledge about health care research. The citizens of Funen were invited to vote for what they believed to be the best research project, and the winning project was awarded 1 million DKK. Our patient representatives participated in the contest through television interviews and publicity to promote the clinical study they were involved in.

#### Interpretation of interim analysis

An interim analysis of the response monitoring stage of the study was performed during November–December 2018. The patient representatives were invited to the meeting where the results from the interim analysis were presented so that they could hear the in-depth explanations of the results and could discuss whether to proceed in the clinical study with or without the blinded PET/CT. The patient representatives were invited to present their opinions and participate in the decision-making.

#### Dissemination of research results

The research group planned the dissemination of study results in patient-friendly language for the participating patients as well as for popular science media. The patient representatives had a central role in these activities.

### Outcome measures: patient recruitment and retention

The *success rate for patient recruitment* (i.e. expected patient recruitment compared to the actual patient recruitment) in the ongoing study with PPI was related to that of the previous study without PPI. For the ongoing study, patient recruitment rates were available at ClinicalTrials.gov [14] and will be published elsewhere in a later phase of the study. For the previous study, data on expected patient recruitment were obtained from the study protocol available at the Department of Nuclear Medicine at Odense University Hospital, while data on observed patient recruitment were obtained from the final publication [13].

For the ongoing study, *patient retention* was assessed 26 months after study initiation. After excluding patients who had left the study due to disease-related issues (e.g. death, comorbidity, side effects), we determined the percentage of patients who continued to attend the regular 3-month follow-up scans. Data on patient retention were not available for the previous study.

We qualitatively observed and described the *researchers’ attitudes towards PPI* during the current study. Not all members of the larger research team participated in the preparation of this manuscript, so we describe here both our own experiences and those observed amongst other members of the research team. The impact of PPI on patient-relevant ethical issues, funding activities, and the dissemination strategy was also observed during the current study.

## Study results

### Major outcomes

#### Patient recruitment and retention

As seen in Table 1, 380 patients were enrolled in the current study as of November 2019 when only 260 patients had been expected, resulting in a patient recruitment of 146% of the expected number. In contrast, 100 of the expected 150 patients were enrolled in the previous study where a 10-month period had to be added, resulting in 67% patient recruitment of the expected number.

**Table 1:** Patient recruitment in two clinical studies of PET/CT for women with advanced breast cancer: the current, ongoing study with patients involved as research partners (PPI) and the previous study without patient involvement

	Expected number of patients (N)	Enrolled number of patients (N)	Fraction of the expected enrollment (%)
Ongoing study^a^	260	380	146
Previous study^b^	150	100	67

^a^with patient and public involvement

^b^without patient and public involvement. The study period was extended with 10 months

Of the 118 women enrolled in the response evaluation stage of the current study (undergoing PET/CT scan every 3 months), approximately 30% (34 patients) had left the study due to disease-related reasons such as death, comorbidity, or side-effects. At the time of analysis, 72 patients (60%) were still active in the study with an average of 5 scans per patient (range 1–9), but 12 patients (10%) had left due to retention issues. Hence, 86% of the eligible patients (72/84) were retained in the study.

#### Patients as partners in planning of the study

The patient representatives found the initially prepared written information overwhelming and confusing for the potential participants. They worried that the written information would cause too much anxiety for the participants at the diagnostic stage when they were informed about the potential long-term outcome if PET/CT revealed an incurable disease. The patient representatives thought that many participants would have difficulties in assessing the impact of the study and would thus decline enrolment.

*“I feared that the patients would black out if the researchers had to inform them upfront about the possible impacts of a PET/CT with metastatic spread (i.e. incurable disease requiring life-long medical treatment). Thus we needed to figure out a better way of informing the patients*”

Patient representative (MLR)

As only one-quarter of the participants in the diagnostic stage of the study were expected to proceed to the response evaluation stage, most participants would receive unnecessary information that could cause anxiety. The outcome of the first meeting was a decision to separate the written information into two parts, one relating to the diagnostic part of the study and the other to the response evaluation. Separate participator information was prepared for and accepted by the larger research team and was later approved by the Danish Ethics Committee.

“*I am so impressed that the patient representative could access the impact of the study so quickly! It is a large and rather complicated study set-up, but the patients just knew how the participants would react if we presented them with the original written information. [They came with] thoughts and feelings that we as researchers would never have considered*.”

Researcher (MV)

The patient representatives shared these positive experiences and were delighted that their contributions to the study had a positive impact on patient recruitment.

### Additional observations on the impact of PPI

#### Involvement in funding activities

The research councils at Odense University Hospital accommodate members of the public and emphasize the importance of PPI in clinical studies carried out at the hospital. We received funding for the large ongoing study, including small acknowledgment fees for the patient representatives.

We believe that patient involvement in the research aspects of the study increased the likelihood of the study being funded.

Although the ongoing study came in second in the funding contest arranged by the university, hospital, and local television, this publicity generated increased awareness of the diagnostic part of the study, and we were afterwards contacted by a few patients living in other regions of Denmark, who requested to participate in the study.

#### Interpretation of interim analysis

The two patient representatives differed in their interpretation of the interim results. While one considered it important to retain the current study design, the other desired a change in the study design due to ethical reasons. Similar differences of opinion were also seen among the other members of the research team.

#### Dissemination of research results

Although results from the ongoing study are not yet available, the patient representatives emphasized the importance of the increased awareness of the study within the patient organization, and this led to other dissemination activities than those initially planned.

“*It is of utmost importance that patients understand the need for them to enroll in health care research in order to improve the treatment of tomorrow*”

Patient representative (MLR)

The two researchers (MV and MGH) were asked to give a presentation on PET/CT and the research study to the local patient organization in September 2017. They were later invited to give two further presentations to the patient organization, one of them at the annual national meeting in March 2018. One of the researchers (MGH) was also interviewed for the patient organization magazine to increase public awareness of the study. Future presentations have been arranged for the patient organization annual meeting in March 2020.

#### Researchers’ attitudes to PPI

Meetings with the patient representatives helped the researchers to better understand women with breast cancer and the inevitable fear of recurrence. The researchers felt that they had learned a lot from the patients, and the initial resistance to PPI within the research team that was driven by concerns, tokenism, and fear of sensitive issues, gradually resolved during the research process.

“*Initially I was concerned about involving patients with previous breast cancer as partners since I find them vulnerable. I worried that they wouldn’t be able to see the perspectives of the study and wondered whether involving them would be worth the time spent. Thankfully, all my concerns were proven wrong*.”

Researcher (MV)

## Discussion

The major outcome from involvement of patients as partners in the research process was a higher than expected patient recruitment in the current study compared to a previous similar study at our institution that did not have patient involvement. The researchers’ attitudes to involving patients as research partners became more positive during the course of the study, and new ideas were generated about how to involve the patient representatives in the dissemination of the research results. However, our results do not allow us to draw any causal conclusions about whether the high patient recruitment and retention rates in the current study were due to PPI alone or in combination with other factors.

The patients who were invited as research partners had previously experienced all steps of the disease from early suspicion of breast cancer to treatment and rehabilitation. These experiences can be categorized as “lived experiences”. Involving such patients as research partners has also led to higher patient enrollment in other studies [4, 5]. The choice of patient representative is important, however. More fragile patients or patients who have not completed treatment and rehabilitation are less likely to be fully able to contribute in the research process.

The greatest impact of PPI in our study was on the development of written information material for the study participants. Contributions from the patient representatives led to major revisions of this material, making it easier to understand and more patient-friendly. Potential participants found it easier to assess the impact of the study and were more likely to accepting enrolment. Such positive effects of PPI are consistent with the results of a recent review, although only 12 studies reported involvement of patients or lay people in the development of patient information material [4].

We experienced and observed a change of attitude of researchers towards involving patients as research partners as the study progressed, as in a learning process. In an early phase of the study, the research team had put much effort into creating optimal participator information material and did not expect any significant input from the patient representatives. This changed, however, as important changes were made to the patient information even in the first meeting with the patients. Patients and researchers have different roles in a research team [4, 8]. The researchers’ role is to ensure high research quality and to justify the research design, while the patients’ role is to bring their perspectives and expertise from lived experience to ensure that the study outcome gives added value for patients [4, 8]. As others have experienced, even the most reluctant researchers in our team applauded the ideas generated from the collaboration with the patient representatives in the later phases of the study [15]. Patient involvement can change both researchers’ actions and their attitudes [9–11].

In the current study, we applied for and received funds for small acknowledgment fees for the patient representatives, who were glad for this contribution to their expenses. Although the need for extra funding is suggested to be a possible barrier for involving patients as research partners [5], we recommend researchers who are planning to involve patients in the research process to apply for additional amounts to cover their travel and other expenses [16–18].

We decided to involve the patient representatives in data interpretation despite concerns about whether they had the necessary scientific knowledge. This resulted in a diverse discussion that was considerably improved by the involvement of the patient representatives. Only sparse knowledge is available in the literature about how and when to involve patients in the more difficult aspects of a research project e.g. data analysis. PPI is most frequently used in the initial phases of a research study rather than in the analysis and dissemination phases [5].

Involvement of the patient representatives led to several presentations to the patient organization. The research team saw this as an important place for disseminating the study results as the organization has an important voice in the public debate. The patient representatives felt obliged to contribute to increased awareness of the study and were very aware of the importance of patients enrolling in clinical trials that aim to improve future treatments.

### Strengths and limitations

A major strength of this study is that we could compare patient recruitment in a study with PPI to that in a previous, similar study at the same institution but without PPI. Although we observed higher patient recruitment in the study with PPI, we cannot claim that this was only due to patient involvement in the research process. Other differences between the two studies must be taken into account. One of these is the timing of the study as the previous study aimed to generate evidence that PET/CT was better for diagnostic purposes than the standard procedures at that time, while for the current study, PET/CT had already been introduced as the new standard procedure for diagnosing advanced breast cancer at our institution. Secondly, the previous study included other scanning modalities and the enrolled patients had more than one scan per day. Thirdly, the current study has a full-time researcher responsible for patient enrolment, whereas in the previous study the researchers in the daily clinic were responsible for enrolling patients. Finally, the current study contains more complex information in the patient material due to the genomic profiling of tumor tissue and blood, requiring decisions about the level of information provided in the case of an inherited genetic disease being detected in the blood sample.

Nevertheless, we are confident that patient involvement in the research team has had a significant and important impact in the current study. The researchers are grateful to these patients and are impressed by their sense of perspective arising from their own experiences with breast cancer.

### Ethical considerations

Involving patients as partners in the research team gave rise to various ethical considerations. The researchers were concerned that the study involvement would cause anxiety for the patients, in view of their previous breast cancer experience, or that they would find it difficult to be objective.

Within the response monitoring stage of the clinical study, ethical considerations arose in relation to blinding of the PET scan as patients typically consider this to be better than a CT scan. Furthermore, the genetic analysis with a possible risk of incidental hereditary findings raised ethical issues.

### Perspectives

Our experience of involving patients as research partners has encouraged us to involve patients at earlier stages in future research studies, e.g. asking patients to develop the written patient information material; having patients as consultants when enrolling patients to the study; inviting patients to help design the study and select patient-relevant endpoints. Patient-reported outcomes and shared decision-making are highly relevant when involving patients as research partners. In addition, it would be interesting to involve patients from the daily clinic as research partners as they may have different views of the research process and outcomes.

Patients should of course not be involved out of tokenism, but with the expectation of a rewarding collaboration between patients and researchers. PPI will clearly not solve all study recruitment issues, but we encourage future health care researchers to involve patients in the research process and to learn from their experiences.

## Conclusion

Involving patients as partners in our research team resulted in major changes to the participator information material and contributed to a higher than expected patient recruitment. We experienced good patient retention with 86% of eligible patients attending for regular follow-up scans. Furthermore, we observed a positive change of attitude amongst the researchers towards patient involvement in the research process.

## Data Availability

All data generated or analyzed during this study are included in this published article [and its supplementary information files].

## Abbreviations

CT: computed tomography
GRIPP-2: guidance for reporting involvement of patients and the public
PET/CT: positron emission tomography/computed tomography
PPI: patient and public involvement
